# EXPRESSION OF CONCERN: Donor Liver Steatosis: A Risk Factor for Early New‐Onset Diabetes after Liver Transplantation

**DOI:** 10.1111/jdi.70350

**Published:** 2026-06-12

**EXPRESSION OF CONCERN**: M. Xue, C. Lv, X. Chen, J. Liang, C. Zhao, Y. Zhang, X. Huang, Q. Sun, T. Wang, J. Gao, J. Zhou, M. Yu, J. Fan, and X. Gao, ‘Donor Liver Steatosis: A Risk Factor for Early New‐Onset Diabetes after Liver Transplantation’, *Journal of Diabetes Investigation* 8, no. 2 (2017): 181–187, https://doi.org/10.1111/jdi.12560.

This Expression of Concern is for the above article, published online on 10 August 2016 in Wiley Online Library (wileyonlinelibrary.com) and has been issued by agreement between the journal Editor‐in‐Chief, Nigishi Hotta; Asian Association for the Study of Diabetes; and John Wiley & Sons Australia, Ltd.

A third‐party organization contacted the publisher regarding concerns pertaining to deceased donor organ sourcing practices in multiple articles, including this one. Credible reporting has been published widely which indicates that research featuring organ transplantation in China did not comply with international ethics standards. As reported by Rogers *et al*., organ donations in China were widely sourced from executed prisoners prior to 2010 [1]. China officially announced an end to the practice of harvesting organs from executed prisoners on January 1, 2015 [2]. Prior to the official end of the practice, a voluntary organ donation program was piloted in select hospitals from 2010 to 2014 [3]. The use of harvested organs from prisoners contravenes ethics guidelines that were in place at the time of publication, including guidelines from the Declaration of Helsinki and the Declaration of Istanbul.

The Materials and Methods for this article indicate that the study “collected hospitalization and outpatient data for patients undergoing liver transplantation between April 2001 and December 2014 at the Liver Transplant Center of Zhongshan Hospital or through clinic follow up and telephone calls.” The article states that the study received approval from the institutional review board of Zhongshan Hospital, Fudan University, and that all participants provided informed consent. No information regarding organ donor sources was provided besides an acknowledgement that the authors reviewed donor type (living and cadaver) and the biopsy results of the donor livers. Because the majority of liver transplant cases reported in this article likely occurred before the existence of a voluntary organ donation program, serious doubts remain regarding the origins of these donations.

The authors did not respond to inquiries made by the publisher. As such, the publisher and editor are not able to verify the institutional review board or ethics committee registration or the informed consent protocols for the study reported in this article. Furthermore, questions remain as to whether the organ donations reported in the article complied with internationally recognized ethical guidelines in place at the time of publication. The journal has decided to issue an Expression of Concern to inform and alert readers.
